# The Association of Stage 1 Hypertension Defined by the 2017 ACC/AHA Guideline with Stroke and Its Subtypes among Elderly Chinese

**DOI:** 10.1155/2020/4023787

**Published:** 2020-02-07

**Authors:** Jinyue Gao, Yue Dai, Yanxia Xie, Jia Zheng, Yali Wang, Rongrong Guo, Zhaoqing Sun, Liying Xing, Xingang Zhang, Yingxian Sun, Liqiang Zheng

**Affiliations:** ^1^Department of Cardiology, Department of Library and Department of Clinical Epidemiology, Shengjing Hospital of China Medical University, Shenyang 110004, China; ^2^Department of Cardiology, Shengjing Hospital of China Medical University, Shenyang 110004, China; ^3^Institute of Chronic Disease, Liaoning Provincial Center for Disease Control and Prevention, Shenyang 110005, China; ^4^Department of Cardiology, The First Affiliated Hospital of China Medical University, Shenyang 110001, China

## Abstract

**Background:**

The 2017 American College of Cardiology and American Heart Association hypertension guideline updated stage 1 hypertension definition as systolic blood pressure range from 130 to 139 mmHg or diastolic blood pressure from 80 to 89 mmHg. However, the association of stage 1 hypertension with stroke and its subtypes among the older population in rural China remains unclear.

**Methods:**

This population-based cohort study consisted of 7,503 adults aged ≥60 years with complete data and no cardiovascular disease at baseline from rural areas of Fuxin County, Liaoning province, China. Follow-up for the new cases of stroke was conducted from the end of the baseline survey to the end of the third follow-up survey (January 1, 2007–December 31, 2017). Adjusted Cox proportional hazards models were used to estimate hazard ratios and 95% confidence intervals with the normal blood pressure as a reference, and calculated population attributable risk was based on prevalence and hazard ratios.

**Results:**

During a median follow-up of 12.5 years, we observed 1,159 first-ever incident stroke (774 ischemic, 360 hemorrhagic, and 25 uncategorized). With the blood pressure <120/<80 mmHg as a reference, stage 1 hypertension showed the adjusted hazard ratios (95% confidence intervals) of 1.45 (1.11–1.90) for all stroke, 1.65 (1.17–2.33) for ischemic stroke, and 1.17 (0.74–1.85) for hemorrhagic stroke, respectively. In this study, the population attributable risk values of stage 1 hypertension were 10.22% (2.64%–18.56%) for all stroke and 14.34% (4.23%–25.41%) for ischemic stroke.

**Conclusion:**

Among adults aged ≥60 years in rural China, stage 1 hypertension defined by 2017 American College of Cardiology and American Heart Association hypertension guideline was independently associated with the increased risk of all stroke and ischemic stroke, excluding hemorrhagic stroke.

## 1. Introduction

Stroke is the leading cause of death in China [1, 2]. In China, recent studies have found that hemorrhagic stroke mortality has decreased; however, morbidity and mortality of hemorrhagic stroke are nearly twice as the global average, and the burden of ischemic stroke is still increasing [3, 4]. Hypertension is a major risk factor for stroke, and the prevalence of hypertension will continue to increase, especially in rural areas [5]. Currently, with the development of economy, the improvement of medical conditions, and the rise in life expectancy, the number of the elderly population in China has increased substantially [6]. The prevalence of hypertension positively increased with age [7, 8]. For this reason, China should pay more attention to blood pressure (BP) in the elderly. In 2017, the American College of Cardiology and American Heart Association (ACC/AHA) released a new guideline, defining systolic/diastolic blood pressure (SBP/DBP) of 130 to 139/80 to 89 mmHg as stage 1 hypertension [9]. Meanwhile, the prevalence of hypertension added under this new definition was concerned by other Asian countries [10–12]. The prevalence of Korean adults with hypertension increased from 28.0% (Seventh Joint National Committee, JNC-7) to 47.9% (2017ACC/AHA guideline) [10]. If China follows the guideline, the prevalence of hypertension will increase from 23.2% to 46.4% and would be higher in the elderly [11].

Many countries in Europe or Asia have carefully considered whether the new guideline is suitable for localization across the difference of geographical regions and ethnicities [13–15]. A data from the Monitoring of Trends and Determinants in Cardiovascular Disease (MONICA)/Cooperative Health Research in the Region of Augsburg (KORA) cohort study involving 11,603 subjects showed that stage 1 hypertension did not increase the risk of cardiovascular disease (CVD) mortality compared with normal BP [13]. Similarly, another study demonstrated that the newly defined stage 1 hypertension was not associated with increased risk of CVD mortality among Chinese in Singapore aged 46–85 years [14]. In contrast, stronger associations with subsequent CVD events were observed in those with baseline stage 1 hypertension among Korean young adults [15]. The findings of previous studies in different countries concerning CVD risk associated with stage 1 hypertension in the new guideline are controversial. These studies did not specifically analyze the relationship between stage 1 hypertension and stroke and its subtypes, especially in rural elderly Chinese.

Therefore, this study aimed to investigate the association between stage 1 hypertension defined by the 2017 ACC/AHA guideline and the risk of stroke and its subtypes among adults aged ≥60 years using the data from rural areas of China.

## 2. Methods

### 2.1. Study Population

The research protocol was approved by the China Medical University Research Ethics Committee, and written informed consent was formally obtained from all the participants or their guardians.

**Figure 1:**
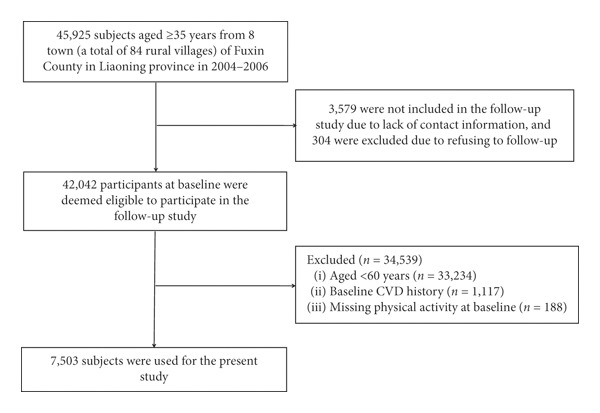
The study population inclusion and exclusion process. CVD: cardiovascular disease.

This is a large-scale epidemiological prospective cohort study. Detailed methods have been described previously [16, 17]. Briefly, from 2004 to 2006, a multistage, random cluster sampling process was used to select a representative sample aged ≥35 years in rural areas of Fuxin County in Liaoning Province, China. All study participants were invited to return for follow-up: from January to July 2008; from July to December 2010; and from March to December 2017. New cases of stroke were collected from the end of the baseline survey to the end of the third follow-up survey (January 1, 2007–December 31, 2017). Of the 45,925 participants at baseline, 3,883 subjects were missing contact information or refused to attend the follow-up, and 42,042 (91.5%) participants were eligible to attend the follow-up at least one time. In the current study, patients meeting the following criteria were excluded: (1) baseline CVD history (stroke and coronary heart disease, including myocardial infarction, arrhythmia, and angina) (*n* = 1,117), (2) missing physical activity at baseline (*n* = 188), and (3) aged < 60 years (*n* = 33,234), leaving 7,503 participants for final analysis. The study population inclusion and exclusion process are illustrated in Figure 1.

### 2.2. Blood Pressure Measurements at Baseline

The BP was measured by a trained research staff according to the American Heart Association protocol. Participants were requested to rest for at least 5 minutes in sitting position and the BP was taken using a standardized automatic electronic sphygmomanometer (HEM-741C; Omron, Tokyo, Japan). Participants were instructed to avoid alcohol consumption, cigarette smoking, coffee/tea, and exercise for at least 30 minutes prior to BP measurement. For each participant, the BP was measured 3 times by trained research staff, and the average of BP values for the current analysis was calculated. According to 2017 ACC/AHA guideline, the participants were divided into four categories: normal (SBP <120 mmHg and DBP <80 mmHg), elevated (120 mmHg ≤SBP ≤129 mmHg and DBP <80 mmHg), stage1 hypertension (130 mmHg ≤SBP ≤139 mmHg or 80 mmHg ≤DBP ≤89 mmHg), and stage 2 hypertension (SBP ≥140 mmHg/DBP ≥90 mmHg or taking antihypertensive medications).

### 2.3. Data Collection

Data on demographic variables (age, sex, and ethnicity), physical examination (height, weight), lifestyle factors (smoking status, alcohol intake, physical activity), history of disease (stroke, coronary heart disease, diabetes, hyperlipidemia, and family history of hypertension), and information on antihypertensive medications were obtained by a trained research staff through standard questionnaire and measurements. Body weight and height were measured with participants wearing light clothing and without shoes. Definitions for body mass index (BMI), smoking, drinking, physical activity, and history of the disease, including stroke, coronary heart disease, diabetes, hyperlipidemia, and family history of hypertension are provided in Supplemental [Supplementary-material supplementary-material-1]. At each follow-up, the information was collected on the occurrence of stroke events and concurrent medication use.

### 2.4. Study Outcomes

The present study outcomes were stroke (fatal and nonfatal). The stroke was defined as rapidly developing signs of focal (or global) disturbance of cerebral function lasting >24 h (unless interrupted by surgery or death) without apparent nonvascular cause, including ischemic, hemorrhage, and uncategorized. The detailed information has been described in the previously published literature [18]. All materials were independently reviewed by the end-point assessment committee, whose members were blinded to the study participants' baseline risk factor information.

### 2.5. Statistical Analysis

Continuous variables were presented as means and standard deviation, while categorical variables were expressed as numbers and percentages. The rates of stroke were presented as the number of events per 1,000 person-years. The baseline participant characteristics were compared using analysis of variance (ANOVA) for continuous variables and Chi-square test was applied for categorical variables.

Adjusted Cox proportional hazard models were used to estimate the association between stage 1 hypertension and stroke; hazard ratios (HRs) and 95% confidence intervals (CIs) were calculated, with the group of normal BP as the reference. Based on the results of univariate analysis (Supplementary [Supplementary-material supplementary-material-1]), reference to clinical practice, and previous researches [19–21], we adjusted the following variables in the 3 models: model 1 was unadjusted; model 2 was adjusted for demographic variables (age, sex, ethnicity, education); and model 3 was adjusted for factors in model 2 and other factors risk of stroke (baseline BMI, current smoking, current drinking, antihypertensive treatment, physical activity, history of diabetes and hyperlipidemia, and family history of hypertension). In addition, the subgroup analysis was analyzed according to sex. The proportional hazards assumption of the Cox models was confirmed using the Schoenfeld residuals. Details on the calculation of population attributable risk (PAR) have been described previously [22]. All analyses were performed by SPSS 22.0 (IBM Inc., Chicago, IL, USA). A 2-sided *P* value less than 0.05 was considered as statistically significant.

## 3. Results

For the present analysis, 7,503 adults aged ≥60 years (mean age: 68.77 ± 6.91 years) were included, and 49.4% were women. The means for SBP and DBP were 144.86 ± 25.58 mmHg and 84.31 ± 13.12 mmHg, respectively. Of these participants, 1,902 (25.3%) were diagnosed as stage 1 hypertension according to the 2017 ACC/AHA hypertension guideline. There were 902 (12.0%) patients taking antihypertensive medications at baseline. Compared with the normal BP level, patients with stage 1 hypertension indicated more Mongolians, more moderate physical activity, and less high physical activity (Table 1).

**Figure 2:**
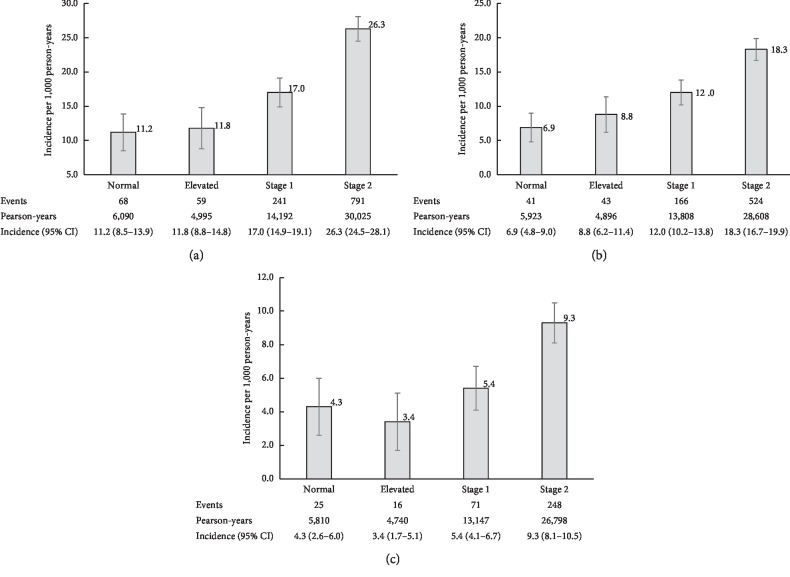
The incidence of stroke and subtypes in newly defined BP levels. (a) All stroke, (b) ischemic stroke, and (c) hemorrhagic stroke. Normal: SBP <120 mmHg and DBP <80 mmHg; elevated: SBP 120–129 mmHg and DBP <80 mm Hg; stage 1, SBP 130–139 mm Hg or DBP 80–89 mm Hg;stage 2, SBP ≥140 mm Hg/DBP ≥90 mm Hg, or taking antihypertensive medications. Error bars represent 95% CI. CI: confidence interval.

During a median follow-up of 12.5 years, we observed 1,159 first-ever strokes (774 ischemic, 360 hemorrhagic, and 25 uncategorized). The number and percentage of new cases of stroke and its subtypes according to BP levels are shown in Supplemental [Supplementary-material supplementary-material-1]. As illustrated in Figure 2, the incidence of stroke (Figure 2(a)) per 1,000 person-years was high in the stage 2 hypertension (26.3; 95% CI: 24.5–28.1), followed by the stage 1 hypertension (17.0; 95% CI: 14.9–19.1), the elevated BP (11.8; 95% CI: 8.8–14.8), and the normal BP (11.2; 95% CI: 8.5–13.9). Similar results were observed in ischemic stroke (Figure 2(b)). However, the incidence of hemorrhagic stroke (Figure 2(c)) was the lowest in the elevated BP (3.4; 95% CI: 1.7–5.1), compared to the normal BP (4.3; 95% CI: 2.6–6.0), stage 1 hypertension (5.4; 95% CI: 4.1–6.7), and stage 2 hypertension (9.3; 95% CI: 8.1–10.5).

Table 2 presents the multivariate Cox proportional hazards models for the risk of stroke relation to different BP levels. After further adjustment by age, sex, ethnicity, education, and other cardiovascular confounders, the result indicated that the HR (95% CI) for all stroke and ischemic stroke were 1.45 (1.11–1.90) and 1.65 (1.17–2.33), respectively (Model 3). There was no statistical evidence for the association between stage 1 hypertension and increased risk of hemorrhagic stroke. Patients with stage 2 hypertension revealed increased risk of all stroke (HR, 2.04; 95% CI: 1.59–2.63), ischemic stroke (HR, 2.35; 95% CI: 1.70–3.25), and hemorrhagic stroke (HR, 1.71; 95% CI: 1.12–2.60), respectively. In women, the HR (95% CI) associated with stage 1 hypertension was 2.83 (1.57–5.10) for all stroke, 2.91 (1.43–5.89) for ischemic stroke, respectively, compared to the normal BP group. This association was not observed in men (Supplementary [Supplementary-material supplementary-material-1]). The results of PAR (95% CI) for all stroke, ischemic stroke, and hemorrhagic stroke associated with stage 1 hypertension were 10.22% (2.64%–18.56%), 14.34% (4.23%–25.41%), and 4.23% (−7.17%–17.99%), respectively.

## 4. Discussion

In the current study, the result showed that patients with stage 1 hypertension have a greater risk of all stroke and ischemic stroke than normal BP participants. However, stage 1 hypertension was not related to an increased hemorrhagic stroke risk compared with normal BP among participants age ≥60 years. In subgroup analysis, compared with normal BP, significantly higher risk of all stroke and ischemic stroke was observed for stage 1 hypertension in women.

The application of stage 1 hypertension in the 2017 ACC/AHA guideline has been a subject of global interest. Many studies investigated the association between stage 1 hypertension and the risk of stroke [15, 23]. A prospective cohort study in the US including 4,851 adults aged 18–30 years showed that before the age of 40 years the HRs of CVD events (including stroke) related to stage 1 hypertension were significantly higher than that related to normal BP [23]. Similarly, an analysis including 2,488,101 participants aged 20–39 years from the population-based cohort study in Korea demonstrated that stage 1 hypertension was associated with a significantly higher risk for stroke compared with normal BP [15]. The above studies' results are in agreement with the present study results; however, the results were limited by the difference of ethnicities, countries, and age groups. Some studies specifically explored the impact of new BP categories on CVD in China [24–26]. Pool analysis results from three prospective cohorts showed that stage 1 hypertension was associated with increased cerebrovascular mortality in the population aged ≥65 years [24]. The Chinese Kailuan Cohort results revealed that stage 1 hypertension presented a significantly higher risk of cerebral infarction and cerebral hemorrhage in the whole population [25]. However, they did not perform age-subgroup analysis. Qi et al. demonstrated that stage 1 hypertension was not correlated with the risk of stroke among the population aged ≥60 years [26]. This finding may be owing to the fact that the number of people aged ≥60 years was small. At the same time, these authors also suggested that further validation using a larger sample size of older people is required. On this basis, our study is supplemental evidence, and we also analyzed the association of stage 1 hypertension with ischemic and hemorrhagic stroke. The Singapore Chinese cohort study showed that there was no association between stage 1 hypertension and an increased risk of stroke mortality [14]. These study participants originate from Fujian and Guangdong provinces in southern China, respectively. The participated population of our study was from the northeast of China. Differences in lifestyle and environmental factors may be the reason for the inconsistent findings of Chinese from different geographical locations of China.

Similar findings were also observed in the previous scholars [27–29]. A meta-analysis found that the prehypertension was associated with a higher risk of ischemic stroke and hemorrhagic stroke in the age group of 30 to 90 years [28]. A prospective cohort study of China demonstrated that an increase of 10 mmHg in SBP (SBP between 120 and 180 mmHg) was associated with 30% increased risk of ischemic stroke and hemorrhagic stroke was about twice as steep among participants aged 40–79 years [29]. These may be due to the differences in the age composition of subjects or BP classification was not in line with stage 1 hypertension.

In this study, several reasons may explain the different risk trends of stroke subtypes. Compared with ischemic stroke cases, the sample size of hemorrhagic stroke may be underpowered for analysis of the association of stage 1 hypertension with hemorrhagic stroke. Ischemic stroke is closely associated with the risk factors for atherosclerosis compared with hemorrhagic stroke [30]. In recent years, living standards in rural areas have improved and increased the intake of cholesterol-rich foods. According to the findings from the China National Stroke Screening and prevention project, the prevalence of dyslipidemia in rural areas has increased compared with previous years [31]. Dyslipidemia, as a risk factor of atherosclerosis, considered as accelerating the process of atherosclerosis [32]. Meanwhile, hypertension is also a risk factor for atherosclerosis [32]. BP has a positive association with atherosclerosis with increasing age [33]. It is suggested that stage 1 hypertension may be more closely related to ischemic stroke in this study. Further studies are required to explore the risk factors for ischemic stroke and hemorrhagic stroke and their relationship with BP. In addition, in subgroup analysis, there was no statistically significant relationship found between stage 1 hypertension and the risk of all stroke and its subtypes in men. In terms of the overall trend, the results of the subgroup analysis were similar to the whole population. In our study, the difference between stage 1 hypertension and stroke risk in men and women is unclear. We hope that more research will focus on this.

Overall, 10.22% of all stroke and 14.34% of ischemic stroke were attributable to stage 1 hypertension. The relatively large PAR observed among elderly participants may be important for stroke prevention. Our findings call for government and health care systems to take public health initiatives to strengthen the management of stage 1 hypertension in the rural elderly Chinese to reduce the incidence of stroke and the burden of stroke on treatment, medical expenditures, and the national economy.

### 4.1. Strength and Limitation

The limitations of this study need to be considered when interpreting the findings. Firstly, although we adjusted for some stroke risk factors in the models, we lacked information about other potential confounding factors (for example, genetic, lifestyle, and environmental factors, blood biochemical data, and renal function etc.), leading to the possibility of residual confounding. In addition, this study is limited to the lack of detailed information on diabetes and cannot perform subgroup analysis by diabetes status, and thus, could not examine the extent to which it might have possible impact on the findings. Secondly, the cases of minor stroke with unclear clinical signs and symptoms may not be recorded owing to poor awareness of timely medical treatment, leading to the underestimation of real stroke cases. Finally, our participants were restricted to rural elderly in northeast China, which limits the generalization of our results. Therefore, further studies are encouraged in different regions and larger older samples to validate the results. Despite these potential limitations, our study presents major strengths, including the large sample size, long period of follow-up (median, 12.5 years), and a large number of stroke cases, which increased the statistical power.

## 5. Conclusion

In conclusion, the present study found that stage 1 hypertension defined by 2017 ACC/AHA hypertension guideline was associated with a higher risk of all stroke and ischemic stroke, but not associated with hemorrhagic stroke among adults aged ≥60 years in rural China. These results imply that the application of the 2017 ACC/AHA hypertension guideline may be beneficial to reduce stroke incidence through lowering BP in stage 1 population.

## Figures and Tables

**Table 1:** Baseline characteristics of study participants aged ≥60 years (*N* = 7,503).

Characteristics	Total (*N* = 7,053)	Blood pressure groups				*P* value
		Normal (*n* = 779)	Elevated (*n* = 636)	Stage 1 (*n* = 1,902)	Stage 2 (*n* = 4,186)	
Age (years)	68.77 ± 6.91	67.76 ± 6.57^d^	68.34 ± 6.52^d^	68.07 ± 6.90	69.35 ± 6.97^a,b^	<0.001
Women, *n* (%)	3,709 (49.4)	384 (49.3)	320 (50.3)^c^	839 (44.1)^b,d^	2,166 (51.7)^c^	<0.001
Ethnicity, *n* (%)						<0.001
Han	5,932 (79.1)	662 (85.0)^b,d^	506 (79.6)^a^	1,537 (80.8)^d^	3,227 (77.1)^a,c^	
Mongolian	1,485 (19.8)	110 (14.1)^c,d^	123 (19.3)	349 (18.3)^a,d^	903 (21.6)^a,c^	
Others	86 (1.1)	7 (0.9)	7 (1.1)	16 (0.8)	56 (1.3)	
Education level, (*n*) %						0.552
Primary school or below	5,832 (77.7)	605 (77.7)	485 (76.3)	1,461 (76.8)	3,281 (78.4)	
Middle school	1,367 (18.2)	145 (18.6)	129 (20.3)	361 (19.0)	732 (17.5)	
High school or above	304 (4.1)	29 (3.7)	22 (3.5)	80 (4.2)	173 (4.1)	
Body mass index (kg/m^2^)						<0.001
<25	6,276 (83.6)	703 (90.2)	576 (90.6)	1,653 (86.9)^d^	3,344 (79.9)^c^	
25∼30	1,101 (14.7)	69 (8.9)^d^	52 (8.2)^c,d^	230 (12.1)^b,d^	750 (17.9)^a,b,c^	
≥30	126 (1.7)	7 (0.9)	8 (1.3)	19 (1.0)^d^	92 (2.2)^c^	
Physical activity, (*n*) %						<0.001
Low	4,917 (65.5)	480 (61.6)	412 (64.8)	1,144 (60.1)^d^	2,881 (68.8)^c^	
Moderate	1,984 (26.4)	200 (25.7)^d^	173 (27.2)	597 (31.4)^d^	1,014 (24.2)^a,c^	
High	602 (8.0)	99 (12.7)^b,c,d^	51 (8.0)^a^	161 (8.5)^a^	291 (7.0)^a^	
Systolic blood pressure (mm Hg)	144.86 ± 25.58	108.65 ± 8.16^b,c,d^	123.98 ± 3.13^a,c,d^	129.81 ± 7.42^a,b,d^	161.61 ± 20.98^a,b,c^	<0.001
Diastolic blood pressure (mm Hg)	84.31 ± 13.12	68.45 ± 6.73^b,c,d^	71.86 ± 5.56^a,c,d^	80.16 ± 5.81^a,b,d^	91.04 ± 12.51^a,b,c^	<0.001
Antihypertensive medications, *n* (%)	902 (12.0)	0 (0.0)	0 (0.0)	0 (0.0)	902 (21.5)	—
Current drinking, *n* (%)	1,898 (25.3)	196 (25.2)	143 (22.5)	454 (23.9)	1,105 (26.4)	0.060
Current smoking, *n* (%)	3,060 (40.8)	320 (41.1)	262 (41.2)	744 (39.1)	1,734 (41.4)	0.396
History of diabetes, *n* (%)	37 (0.5)	2 (0.3)	3 (0.5)	7 (0.4)	25 (0.6)	0.489
History of hyperlipidemia, *n* (%)	261 (3.5)	12 (1.5)^d^	10 (1.6)^d^	36 (1.9)^d^	203 (4.8)^a,b,c^	<0.001
Family history of hypertension, *n* (%)	366 (4.9)	17 (2.2)^d^	12 (1.9)^d^	51 (2.7)^d^	286 (6.8)^a,b,c^	<0.001

Values are mean ± SD or *n* (%). *P* values from ANOVA for continuous variables; normal: <120/80 mm Hg; Elevated:120–129/<80 mm Hg; stage 1: 130–139/80–89 mm Hg; stage 2: ≥140/90 mm Hg or accepted antihypertensive treatment; ^a^ vs. normal *P* < 0.05; ^b^ vs. elevated *P* < 0.05; ^c^ vs. stage 1 *P* < 0.05; ^d^ vs. stage 2 *P* < 0.05.

**Table 2:** Cox proportional hazards models and the PARs (%) for stroke and subtypes. (*N* = 7,503).

SBP/DBP categories (mm Hg)	Stroke			Ischemic stroke			Hemorrhagic stroke		
	HR (95% CI)	*P* value	PAR (95% CI), %	HR (95% CI)	*P* value	PAR (95% CI), %	HR (95% CI)	*P* value	PAR (95% CI), %
Model 1^a^									
<120/<80	1.00 (ref)			1.00 (ref)			1.00 (ref)		
120–129/<80	1.05 (0.74–1.49)	0.779	1.28 (−7.00–11.05)	1.26 (0.82–1.94)	0.287	6.28 (−4.79–19.35)	0.78 (0.42–1.46)	0.440	−6.00 (−17.73–10.68)
130–139/80–89	1.54 (1.17–2.01)	**0.002**	12.00 (4.22–20.40)	1.76 (1.25–2.47)	**0.001**	16.23 (5.94–27.39)	1.26 (0.80–1.99)	0.315	6.36 (−5.42–20.40)
≥140/≥90	2.43 (1.90–3.12)	**<0.001**	26.62 (18.54–34.90)	2.74 (1.99–3.76)	**<0.001**	30.81 (20.26–41.46)	2.18 (1.44–3.29)	**<0.001**	23.32 (10.27–37.13)

Model 2^b^									
<120/<80	1.00 (ref)			1.00 (ref)			1.00 (ref)		
120–129/<80	1.03 (0.73–1.46)	0.859	0.80 (−7.41–10.50)	1.23 (0.80–1.88)	0.349	5.51 (−5.41–18.46)	0.77 (0.41–1.43)	0.403	−6.46 (−18.06–10.06)
130–139/80–89	1.47 (1.12–1.92)	**0.005**	10.65 (3.02–18.98)	1.67 (1.18–2.34)	**0.004**	14.58 (4.46–25.65)	1.20 (0.76–1.90)	0.431	4.94 (−6.58–18.78)
≥140/≥90	2.30 (1.79–2.95)	**<0.001**	24.74 (16.72–33.02)	2.60 (1.89–3.57)	**<0.001**	29.09 (18.58–39.77)	1.99 (1.32–3.01)	**0.001**	20.40 (7.61–34.18)

Model 3^c^									
<120/<80	1.00 (ref)			1.00 (ref)			1.00 (ref)		
120–129/<80	1.03 (0.72–1.46)	0.885	0.65 (−7.52–10.34)	1.22 (0.80–1.88)	0.357	5.41 (−5.50–18.37)	0.75 (0.40–1.41)	0.377	−6.82 (−18.27–9.62)
130–139/80–89	1.45 (1.11–1.90)	**0.007**	10.22 (2.64–18.56)	1.65 (1.17–2.33)	**0.004**	14.34 (4.23–25.41)	1.17 (0.74–1.85)	0.500	4.23 (−7.17–17.99)
≥140/≥90	2.04 (1.59–2.63)	**<0.001**	20.88 (12.91–29.23)	2.35 (1.70–3.25)	**<0.001**	25.76 (15.27–36.62)	1.71 (1.12–2.60)	**0.013**	15.43 (2.96–29.30)

CI = confidence interval, DBP = diastolic blood pressure, HR = hazard ratios, PAR = population attributable risk, SBP = systolic blood pressure. ^a^Model 1: unadjusted. ^b^Model 2: adjusted for age, sex, ethnicity, and education. ^c^Model 3: adjusted for age, sex, ethnicity, education, body mass index, smoking, drinking, antihypertension treatment, physical activity, history of diabetes and hyperlipidemia, and family history of hypertension.

## Data Availability

The data used to support the findings of this study are available from the corresponding author upon request.

## Supplementary Materials

Supplementary Materials Supplementary Table 1 shows definitions for baseline characteristics, including BMI, currently smoking, current drinking, physical activity, and history of the disease. Body mass index was calculated as weight in kilograms divided by square of height in meters. Smoking was defined as currently smoking at least one cigarette per day and lasting for at least a year. Current drinking was defined as men and women were consuming beer, wine, or liquor at least 2 cups/day and at least 1 cup/day, respectively. Based on the occupational and leisure-time physical activity, the physical activity was recategorized into three levels: low, moderate, and high. A self-reported history of medical conditions diagnosed by physicians, including stroke, coronary heart disease, diabetes, and hyperlipidemia, was collected. Family history of hypertension was defined as one of their parents was with hypertension. Supplementary Table 2 demonstrates that the results of univariate Cox proportional hazards models. Based on the results of the univariate analysis, we included age (*P* < 0.001), sex (*P* < 0.001), ethnicity (*P*=0.114), education (*P*=0.025), physical activity (*P*=0.031), current drinking (*P*=0.070), history of hyperlipidemia (*P*=0.049), and antihypertensive treatment (*P* < 0.001) into the models. *P* value less than 0.15 was accepted as statistically significant. Supplementary Table 3 presents the number and percentage of new cases of stroke and its subtypes according to blood pressure levels. The number and proportions of all stroke events were 68 (5.9), 59 (5.1), 241 (20.8), and 791 (68.2), of ischemic stroke cases were 41 (5.3), 43 (5.6), 166 (21.4), 524 (67.7) and of hemorrhagic stroke cases were 25 (6.9), 16 (4.4), 71 (19.7), 248 (68.9) in four BP classification of normal BP, elevated BP, stage 1 hypertension, and stage 2 hypertension, respectively. Supplementary Table 4 shows HRs (95% CI) for the associations between BP levels and the risk of stroke stratified by sex. Women with stage 1 hypertension revealed increased risk of all stroke (HR, 2.83; 95% CI: 1.57–5.10), ischemic stroke (HR, 2.91; 95% CI: 1.43–5.89), respectively. In contrast, the association was not observed in men: the HRs (95% CI) for all stroke and ischemic stroke were 1.12 (0.82–1.52) and 1.29 (0.87–1.92), respectively. Meanwhile, compared with normal BP, a significantly higher risk of hemorrhagic stroke was not observed for stage 1 hypertension in women (HR, 2.59; 95% CI: 0.89–7.54) and men (HR, 0.88; 95% CI: 0.52–1.47).
