# Supplementary material for: The Association of Stage 1 Hypertension Defined by the 2017 ACC/AHA Guideline with Stroke and Its Subtypes among Elderly Chinese
Source: Biomed Res Int. 2020 Feb 7;2020:4023787. doi: 10.1155/2020/4023787 (PMC7035505; doi:10.1155/2020/4023787)
Supplement: Supplementary Materials — Supplementary Table 1 shows definitions for baseline characteristics, including BMI, currently smoking, current drinking, physical activity, and history of the disease. Body mass index was calculated as weight in kilograms divided by square of height in meters. Smoking was defined as currently smoking at least one cigarette per day and lasting for at least a year. Current drinking was defined as men and women were consuming beer, wine, or liquor at least 2 cups/day and at least 1 cup/day, respectively. Based on the occupational and leisure-time physical activity, the physical activity was recategorized into three levels: low, moderate, and high. A self-reported history of medical conditions diagnosed by physicians, including stroke, coronary heart disease, diabetes, and hyperlipidemia, was collected. Family history of hypertension was defined as one of their parents was with hypertension. Supplementary Table 2 demonstrates that the results of univariate Cox proportional hazards models. Based on the results of the univariate analysis, we included age (P < 0.001), sex (P < 0.001), ethnicity (P=0.114), education (P=0.025), physical activity (P=0.031), current drinking (P=0.070), history of hyperlipidemia (P=0.049), and antihypertensive treatment (P < 0.001) into the models. P value less than 0.15 was accepted as statistically significant. Supplementary Table 3 presents the number and percentage of new cases of stroke and its subtypes according to blood pressure levels. The number and proportions of all stroke events were 68 (5.9), 59 (5.1), 241 (20.8), and 791 (68.2), of ischemic stroke cases were 41 (5.3), 43 (5.6), 166 (21.4), 524 (67.7) and of hemorrhagic stroke cases were 25 (6.9), 16 (4.4), 71 (19.7), 248 (68.9) in four BP classification of normal BP, elevated BP, stage 1 hypertension, and stage 2 hypertension, respectively. Supplementary Table 4 shows HRs (95% CI) for the associations between BP levels and the risk of stroke stratified by sex. Women with stage 1 [file 4023787.f1.docx]

| **Supplemental Table 1. Definitions for baseline characteristics.** | | |
| --- | --- | --- |
| Baseline characteristics |  | Definitions |
| Body mass index |  | Body mass index was calculated as weight in kilograms divided by  square of height in meters. |
| Smoking |  | Smoking was defined as currently smoking at least one cigarette per  day and lasting for at least a year. |
| Current drinking |  | Current drinking was defined as men and women were consuming beer, wine or liquor at least 2 cups/day and at least 1 cup/day respectively. |
| Physical activity |  | Based on the occupational and leisure-time physical activity, the physical activity were re-categorized into three levels: low, moderate and high. |
| History of the disease |  | A self-reported history of medical conditions diagnosed by physicians, including stroke, coronary heart disease, diabetes and hyperlipidemia, was collected. |
|  |  | Family history of hypertension was defined as one of their parents with hypertension. |

| **Supplementary Table 2. The results of univariate analysis.** | | |
| --- | --- | --- |
| Variables | HR (95% CI) | *P*-Value |
| Age | 1.06 (1.05-1.06) | **<0.001** |
| Sex | 0.63 (0.56-0.71) | **<0.001** |
| Ethnicity | 1.11 (0.98-1.26) | **0.114** |
| Education | 0.88 (0.78-0.98) | **0.025** |
| Body mass index | 1.00 (0.88-1.15) | 0.971 |
| Current smoking | 1.07 (0.95-1.20) | 0.251 |
| Current drinking | 1.13 (0.99-1.28) | **0.070** |
| Antihypertension treatment | 1.72 (1.48-2.00) | **<0.001** |
| Physical activity | 1.11 (1.01-1.23) | **0.031** |
| History of diabetes | 1.19 (0.49-2.86) | 0.700 |
| History of hyperlipidemia | 1.32 (1.00-1.74) | **0.049** |
| Family history of hypertension | 1.18 (0.93-1.51) | 0.178 |
| *P* value less than 0.15 was accepted as statistically significant. | | |

**Supplementary Table 3. The number and percentage of new cases of stroke and its subtypes according to blood pressure levels.**

| Events |  | Blood pressure groups | | | |
| --- | --- | --- | --- | --- | --- |
|  | Total | Normal | Elevated | Stage 1 | Stage 2 |
|  | (N=7503) | (n=779) | (n=636) | (n=1,902) | (n=4,186) |
| All stroke, n (%) | 1159 | 68 (5.9) | 59 (5.1) | 241 (20.8) | 791 (68.2) |
| Ischemic stroke, n (%) | 774 | 41 (5.3) | 43 (5.6) | 166 (21.4) | 524 (67.7) |
| Hemorrhagic stroke, n (%) | 360 | 25 (6.9) | 16 (4.4) | 71 (19.7) | 248 (68.9) |

Normal: <120/80 mm Hg; Elevated: 120-129/<80 mm Hg; Stage 1:130-139/80-89 mm Hg; Stage 2: ≥140/90 mm Hg or accepted antihypertensive treatment.

**Supplementary Table 4.** **Multivariate** **Cox proportional hazards models for stroke and its subtypes stratified by sex*****. (N=7,503)**

| SBP/DBP categories (mm Hg) | Stroke | |  | Ischemic Stroke | | | Hemorrhagic Stroke | | | |
| --- | --- | --- | --- | --- | --- | --- | --- | --- | --- | --- |
|  | HR (95% CI) | *P* value |  |  | HR (95% CI) | *P* value |  |  | HR (95% CI) | *P* value |
| Men |  |  |  |  |  |  |  |  |  |  |
| <120/<80 | 1.00 (Ref) |  |  |  | 1.00 (Ref) |  |  |  | 1.00 (Ref) |  |
| 120-129/<80 | 0.74 (0.48-1.13) | 0.165 |  |  | 0.83 (0.49-1.42) | 0.504 |  |  | 0.61 (0.30-1.28) | 0.191 |
| 130-139/80-89 | 1.12 (0.82-1.52) | 0.487 |  |  | 1.29 (0.87-1.92) | 0.203 |  |  | 0.88 (0.52-1.47) | 0.615 |
| ≥140/≥90 | 1.55 (1.16-2.06) | **0.003** |  |  | 1.87 (1.29-2.71) | **0.001** |  |  | 1.14 (0.71-1.83) | 0.592 |
| Women |  |  |  |  |  |  |  |  |  |  |
| <120/<80 | 1.00 (Ref) |  |  |  | 1.00 (Ref) |  |  |  | 1.00 (Ref) |  |
| 120-129/<80 | 2.23 (1.14-4.36) | **0.019** |  |  | 2.57 (1.17-5.65) | **0.019** |  |  | 1.51 (0.40-5.63) | 0.541 |
| 130-139/80-89 | 2.83 (1.57-5.10) | **0.001** |  |  | 2.91 (1.43-5.89) | **0.003** |  |  | 2.59 (0.89-7.54) | 0.080 |
| ≥140/≥90 | 4.08 (2.33-7.15) | **<0.001** |  |  | 4.05 (2.06-7.95) | **<0.001** |  |  | 4.57 (1.66-12.55) | **0.003** |

CI=confidence interval, DBP=diastolic blood pressure, HR=hazard ratios, PAR= population attributable risk, SBP=systolic blood pressure.
*adjusted for age, ethnicity, education, body mass index, smoking, drinking, antihypertension treatment, physical activity, history of diabetes and hyperlipidemia, and family history of hypertension.
